# Characteristics and comparative clinical outcomes of prisoner versus non-prisoner populations hospitalized with COVID-19

**DOI:** 10.1038/s41598-021-85916-w

**Published:** 2021-03-22

**Authors:** Ahmed M. Altibi, Bhargava Pallavi, Hassan Liaqat, Alexander A. Slota, Radhika Sheth, Lama Al Jebbawi, Matthew E. George, Allison LeDuc, Enas Abdallah, Luke R. Russell, Saniya Jain, Nariné Shirvanian, Ahmad Masri, Vivek Kak

**Affiliations:** 1 Department of Internal Medicine, Henry Ford Allegiance Hospital, Henry Ford Health System, 205 N East Ave, Jackson, MI 49201 USA; 2 Harvard T.H. Chan School of Public Health, Harvard University, Boston, MA USA; 3 Department of Internal Medicine, Division of Infectious Diseases, Henry Ford Hospital, Detroit and West Bloomfield, MI 48322 USA; 4 Division of Hospital Medicine, Henry Ford West Bloomfield, West Bloomfield, MI 48322 USA; 5 Michigan State University College of Osteopathic Medicine, Lansing, MI 48824 USA; 6 Knight Cardiovascular Institute, Oregon Health and Science University, Portland, OR 97239 USA

**Keywords:** Diseases, Medical research, Risk factors

## Abstract

Prisons in the United States have become a hotbed for spreading COVID-19 among incarcerated individuals. COVID-19 cases among prisoners are on the rise, with more than 143,000 confirmed cases to date. However, there is paucity of data addressing clinical outcomes and mortality in prisoners hospitalized with COVID-19. An observational study of all patients hospitalized with COVID-19 between March 10 and May 10, 2020 at two Henry Ford Health System hospitals in Michigan. Clinical outcomes were compared amongst hospitalized prisoners and non-prisoner patients. The primary outcomes were intubation rates, in-hospital mortality, and 30-day mortality. Multivariable logistic regression and Cox-regression models were used to investigate primary outcomes. Of the 706 hospitalized COVID-19 patients (mean age 66.7 ± 16.1 years, 57% males, and 44% black), 108 were prisoners and 598 were non-prisoners. Compared to non-prisoners, prisoners were more likely to present with fever, tachypnea, hypoxemia, and markedly elevated inflammatory markers. Prisoners were more commonly admitted to the intensive care unit (ICU) (26.9% vs. 18.7%), required vasopressors (24.1% vs. 9.9%), and intubated (25.0% vs. 15.2%). Prisoners had higher unadjusted inpatient mortality (29.6% vs. 20.1%) and 30-day mortality (34.3% vs. 24.6%). In the adjusted models, prisoner status was associated with higher in-hospital death (odds ratio, 2.32; 95% confidence interval (CI), 1.33 to 4.05) and 30-day mortality (hazard ratio, 2.00; 95% CI, 1.33 to 3.00). In this cohort of hospitalized COVID-19 patients, prisoner status was associated with more severe clinical presentation, higher rates of ICU admissions, vasopressors requirement, intubation, in-hospital mortality, and 30-day mortality.

## Introduction

In 2016, there were nearly 2.2 million incarcerated individuals in U.S correctional facilities. Incarcerated individuals are among the most vulnerable for the outspread of infectious diseases due to restricted mobility, overcrowding, confined spaces, limited resources and inequitable access to healthcare services^1, 2^. The first known case of Coronavirus Disease 2019 (COVID-19) in a U.S prison occurred in mid-March at the Rikers Island Correctional Facility of New York City. Within few weeks, over 200 cases were diagnosed despite all efforts to curb its spread within the facility^2^. Since then, the outspread of COVID-19 in U.S prisons continued to rampage, with no sign of a respite^3^. As of October 6, 2020, at least 143,243 inmates in U.S. prisons were reported to have tested positive for COVID-19 and 1210 died^3^. Nationwide estimates for COVID-19 infection rates among prisoners are five times higher than in general population^3, 4^. Despite this mortifying public health crisis, current federal and state-level healthcare policies failed to respond adequately in a manner that would lead to “flattening the curve” in U.S prisons^1^.

In the absence of an effective vaccine, physical distancing strategies remain the most effective mitigative measure to halt the rate of COVID-19 spread. Such strategies were demonstrated to be effectual during the Spanish influenza pandemic when early, sustained, and sweeping imposition of public gathering restrictions in the 1918 managed to cease the flu’s death toll in cities across America^5^. Yet, in situations where practicing physical distancing was not attainable, infection rates and mortality statistics can be astounding—as was seen on cruise ships (*Diamond Princes* cruise ship) and nursing home facilities^6^. Similarly, physical distancing is practically impossible in the setting of incarceration unless major shift in policies occurs. This challenge has led some local authorities in the U.S to adopt depopulation measures to decrease the number of incarcerated individuals in prisons.

The State of Michigan has been a hotbed for COVID-19 spread among prisoners. With a total of 24,000 documented cases as of January 2021, Michigan ranks third across the nation with the number of COVID-19 cases per 10,000 prisoners^3^. Michigan Department of Correction has adopted a strategy of universal nasopharyngeal swab testing for Severe Acute Respiratory Syndrome Coronavirus-2 (SARS-CoV-2) for all prisoners aiming to identify asymptomatic carriers. By the end of May, 2020, a total of 38,130 prisoners have been tested and 3,982 (10.4%) were positive for SARS-CoV-2^7^. Clinical data about outcomes of prisoners with COVID-19 are lacking. In this study, we sought to report on the characteristics and clinical outcomes of prisoners hospitalized with COVID-19 as compared to non-prisoners.

## Methods

### Study design, settings, and population

This was a retrospective cohort study of all consecutive patients hospitalized between March 10 and May 10, 2020 and tested positive for SARS-CoV-2 on qualitative polymerase chain-reaction (PCR) assay at two Henry Ford Health System hospitals. Patients evaluated at the emergency department without being hospitalized were excluded. The first admission with COVID-19 was considered as the index admission; data was extracted from the index admission while data from readmissions were disregarded. The study was reviewed and approved by Henry Ford Health System (HFHS) Institutional Review Board (IRB). Patients included in the study were those hospitalized at two Henry Ford hospitals (i) Henry Ford Allegiance Health: 475-bed hospital located in Jackson county, Michigan and (ii) Henry Ford West Bloomfield: 200-bed hospital located in Oakland county, Michigan. Admission criteria and treatment protocols were similar at both hospitals based upon healthcare system’s COVID-19 management guidelines.

### Data collection

We collected patient-level data on imprisonment status (prisoner versus non-prisoner), demographic characteristics (age, sex, and race), chronic medical conditions, smoking status, and obesity (determined by body-mass index). Additional information included patient-reported symptoms, vital signs, medications, laboratory findings, complications, and treatment outcomes. Reported clinical data were linked to inpatient encounters during which a patient tested positive for COVID-19, or first inpatient encounters that followed an out-of-hospital positive COVID-19 test. Upon hospitalization, COVID-19 testing was repeated to confirm diagnosis when initial positive COVID-19 testing could not be documented. Follow-up information was obtained by contacting patients, families, or nursing home facilities via phone. Prisoners’ follow-up information was obtained by contacting the healthcare staff at the prison facilities.

### Outcome measures

The prespecified primary outcomes for the study were in-hospital mortality, 14-day mortality and 30-day mortality in both prisoner and non-prisoner groups. Secondary outcomes include ICU admission rates, intubation rates, length of hospital stay (LOS), disposition status at discharge (home, nursing facility, or hospice home), and 30-day readmission rate for both prisoners and non-prisoners.

### Statistical analysis

Characteristics of COVID-19 patients were compared according to imprisonment status (prisoners vs. non-prisoners). For continuous variables, measures are presented as means and standard deviations (SD) or medians and interquartile ranges (IQRs). Categorical measures are presented as percentages. Each outcome measure was assessed using unadjusted and adjusted models. Factors associated with in-patient mortality and intubation were examined with the use of multivariable logistic regression models. Factors associated with 30-day mortality were investigated with the Cox proportional-hazards models. Each of the primary outcomes was examined using three models: (i) model 1: included imprisonment status only, (ii) model 2: included imprisonment status, age, and sex, (iii) model 3: included model 2 variables, obesity, and Charlson Comorbidity Index (CCI) score. CCI score was calculated on the basis of 17 comorbid conditions, each of which is assigned a weighted score^8^. A higher score on the CCI indicates a greater comorbidity burden. All model covariates were selected a priori based on clinical relevance, demonstrated association with clinical outcomes in previous studies, or bivariate analyses with outcomes.

Data for intubation and in-hospital mortality were complete for the whole cohort; while data for 30-day mortality was missing for 9 patients, all from the non-prisoner group. Patients with missing 30-day mortality were not included in the Cox proportional-hazards models. Missing data for covariates were handled with the use of multiple imputation. Multiple imputation models incorporated all available baseline data. Results from the analyses of inpatient mortality and intubation are reported as odds ratios and results from the analyses of 30-day mortality are reported as hazard ratios. The proportional-hazards assumption for Cox models was investigated based on Schoenfeld residual method as well as graphically. Survival curves were plotted using the Kaplan–Meier method to compare 30-day mortality between the two groups. Log-Rank test was used to test equality of survival functions. A *p* value < 0.05 was considered statistically significant. All statistical analyses were performed with the use of STATA 16 (State Corp LLC, College Station, Texas).

## Results

### Characteristics of the cohort

The first case of COVID-19 among prisoners in Michigan was identified on March 22, 2020; while the first hospitalization for a prisoner in our hospitals was on March 25, 2020. A total of 706 consecutive patients with COVID-19 were included in the analyses. Of them, 108 patients (15.3%) were prisoners and 598 were non-prisoners. The mean age of patients in the cohort was 66.7 ± 16.1 years, 56.5% were males (98.0% of prisoners were males), 44.3% were Black and 48.0% were Caucasians. Overall, prisoners had a higher CCI score (1.85 versus 1.57) and a higher prevalence of COPD, and underlying malignancies. While, non-prisoners were older (67.7 vs. 61.2 years) and had a higher prevalence of chronic kidney disease, obesity, and dementia. Comorbidities in both groups are detailed in Table 1.

**Table 1:** Demographics and baseline characteristics for hospitalized COVID-19 positive patient, comparing prisoners to non-prisoners.

	Prisoners (N = 108)	Non-prisoners (N = 598)	*p* value
Male, n. (%)	106 (98.2%)	293 (49.0%)	< **0.001**
Age, years^a^	61.2 ± 12.5	67.7 ± 16.5	< **0.001**
Age > 65 years	40 (37.0%)	337 (56.4%)	< **0.001**
Body mass index (BMI), kg/m^2^	29.5 ± 5.8	30.4 ± 7.9	0.15
Obesity (BMI ≥ 30)	45 (41.7%)	276/595 (46.4%)	0.37
Morbid obesity (BMI ≥ 40)	5 (4.6%)	70/595 (11.8%)	**0.03**
Race^a^			
Caucasian	47 (43.5%)	285/583 (48.9%)	0.31
Black	54 (50.0%)	252/583 (43.2%)	
Others	7 (6.5%)	46/583 (7.9%)	
Smoking status^a^			
Non-smoker	19/96 (19.8%)	355/569 (62.4%)	< **0.001**
Active smoker	3/96 (3.1%)	25/569 (4.4%)	
Former smoker	74/96 (77.1%)	189/569 (33.2%)	
Hypertension^b^	76 (70.4%)	438 (73.2%)	0.54
Dyslipidemia^b^	54 (50.0%)	347 (58.0%)	0.12
Coronary artery disease^b^	20 (18.5%)	117 (19.6%)	0.80
History of myocardial infarction^b^	15 (13.9%)	52 (8.7%)	0.09
History of congestive heart failure^b^	16 (14.8%)	98 (16.4%)	0.68
Peripheral vascular disease^b^	9 (8.3%)	38 (6.4%)	0.45
Cerebrovascular disease^**b**^	10 (9.3%)	70 (11.7%)	0.46
Chronic liver disease^b^	2 (1.9%)	14 (2.3%)	0.75
Chronic kidney disease^b^	11 (10.2%)	143 (23.9%)	**0.002**
Dialysis dependent	1 (0.9%)	14 (2.3%)	0.35
Kidney transplant	0 (0.0%)	6 (1.0%)	0.30
Type 2 diabetes mellitus (DM)^b^	39 (36.1%)	199 (33.3%)	0.53
Insulin-dependent DM	14 (12.9%)	51 (8.5%)	0.14
Complicated DM*	5 (4.6%)	68 (11.4%)	**0.04**
Malignancy^b^	10 (9.3%)	22 (3.7%)	**0.01**
Solid tumors	8 (7.4%)	15 (2.5%)	**0.01**
Leukemia/lymphoma	3 (2.8%)	7 (1.2%)	0.13
Dementia^b^	2 (1.9%)	108 (18.1%)	< **0.001**
Hemiplegia^b^	1 (0.9%)	16 (2.7%)	0.28
HIV^b,^^c^	3 (2.8%)	3 (0.5%)	**0.02**
Hepatitis C^b^	4 (3.7%)	6 (1.0%)	**0.03**
Chronic lung disease^b^			
COPD^b,^^c^	40 (37.0%)	63 (10.5%)	< **0.001**
Asthma	10 (9.3%)	65 (10.9%)	0.61
Sleep apnea	6 (5.6%)	73 (12.2%)	**0.04**
Interstitial lung disease	0 (0.0%)	7 (1.2%)	0.26
Pulmonary sarcoidosis	0 (0.0%)	5 (0.8%)	0.34
Oxygen dependent	6 (5.6%)	25 (4.2%)	0.52
CCI score	1.85 ± 2.02	1.57 ± 1.79	0.17

Bold values indicates clinically significant *p* values (*p* value < 0.05).

^a^Plus–minus values indicate means ± standard deviation (SD). Data for race and smoking status were missing for 15 and 41 patients, respectively. Race and smoking status were self-reported by the patients.

^b^Absence of diagnoses recorded in the medical record was assumed to mean absence of the conditions.

^c^HIV denotes Human Immunodeficiency Virus; COPD denotes chronic obstructive pulmonary disease.

### Clinical signs and laboratory findings

The median time from self-reported onset of symptoms to admission was 6 (IQR: 3–8) days for prisoners and 5 (IQR: 2–8) days for non-prisoners. Compared to non-prisoners, prisoners had worse clinical signs at presentation, including fever (temperature ≥ 38.0 °C), hypoxemia (oxygen saturation < 94%), and tachypnea (respiratory rate > 24/minute). Further, prisoners had markedly higher levels of inflammatory markers upon admission, including CRP, ferritin, and LDH. Clinical signs and laboratory findings upon admission and during hospital course are detailed in Table S1.

### Medical treatment and hospital course

During hospital course, 25.6% of patients did not require oxygen support, 41.6% required oxygen support delivered via conventional nasal cannula, 16.4% required high-flow nasal cannula, and 19.9% required intubation. Treatment protocol included anticoagulation (84.0%), corticosteroids (79.4%), antibiotics (46.2%) for prevention/treatment of secondary infections, and hydroxychloroquine (64.8%). Subcutaneous enoxaparin was the anticoagulant of choice in most patients (73%). Corticosteroids were in the form of intravenous methylprednisolone (1–2 mg/kg/day). Tocilizumab, an interleukin-6 inhibitor, was given to 7.2% of patients at a dose of 400 mg intravenously based on clinic criteria. Other treatment modalities include Remdesivir (compassionate use protocol) and Convalescent Plasma (part of Mayo Clinic’s Expanded Access to Convalescent Plasma trial)^9^. Details of treatment modalities are shown in Table 2. Complications accompanying hospital course are shown in Table S2 (Supplement 1).

**Table 2:** Treatment, complications, and outcomes in patients hospitalized with COVID-19 (prisoners vs. non-prisoners).

	Prisoners (N = 108)	Non-prisoners (N = 598)	*p* value
**Therapeutics and medications**			
Highest O_2_ requirement			
Ambient air	16 (14.8%)	150 (25.1%)	**0.02**
Nasal cannula	23 (21.3%)	271 (45.3%)	< **0.001**
High-flow nasal cannula	38 (35.2%)	78 (13.0%)	< **0.001**
NIVM^a^	4 (3.7%)	7 (1.2%)	0.05
IMV^a^	27 (25.0%)	92 (15.4%)	**0.01**
Antibiotics	55 (50.9%)	271 (45.3%)	0.28
Corticosteroids	95 (88.0%)	466 (78.0%)	**0.02**
Anticoagulation	103 (95.4%)	490 (81.9%)	< **0.001**
Hydroxychloroquine	49 (45.4%)	409 (68.4%)	< **0.001**
Tocilizumab	17 (15.7%)	34 (5.7%)	< **0.001**
Remdesivir	2 (1.9%)	3 (0.5%)	0.81
Convalescent plasma	9 (8.3%)	7 (1.2%)	< **0.001**
Renal replacement therapy	6 (5.6%)	24 (4.0%)	0.46
Vasopressors	26 (24.1%)	59 (9.9%)	< **0.001**
ECMO^a^	0 (0.0%)	2 (0.3%)	0.55
**Clinical outcomes**			
Length of stay (days)	9 (5–14)	7 (5–12)	0.09
ICU admission^a^	29 (26.9%)	112 (18.7%)	0.05
Intubation	27 (25.0%)	91 (15.2%)	**0.01**
Inpatient mortality	32 (29.6%)	120 (20.1%)	**0.03**
14-day mortality^b^	21 (19.4%)	102 (17.1%)	0.60
30-day mortality^b^	37 (34.3%)	145 (24.6%)	**0.04**
30-day readmission^b^			
Readmitted	7/76 (9.2%)	65/478 (13.6%)	0.053
Not readmitted	69/76 (90.8%)	387/478 (81.0%)	
Unknown	0/76 (0.0%)	26/478 (5.4%)	
Disposition status^c^			
Home	–	363 (75.8%)	–
Other facilities	–	96 (20.0%)	
Hospice home	–	20 (4.2%)	

Bold values indicates clinically significant *p* values (*p* value < 0.05).

^a^NIVM indicates non-invasive mechanical ventilation; IMV indicates invasive mechanical ventilation; ECMO indicates extracorporeal mechanical oxygenation; ICU indicates intensive care unit.

^b^14-day mortality and 30-day mortality data were missing for 10 patients from the non-prisoner group. 30-day readmission data was missing for 26 patients from the non-prisoner group. Only patients who were discharged alive were included in calculating the 30-day readmission rate.

^c^Disposition status percentages were calculated from the total number of patients discharged alive (479 in the non-prisoner group). Other facilities include skilled nursing facilities and rehabilitation facilities (short-term, long-term, and inpatient rehabilitation).

### Mortality outcomes

**Figure 1:**
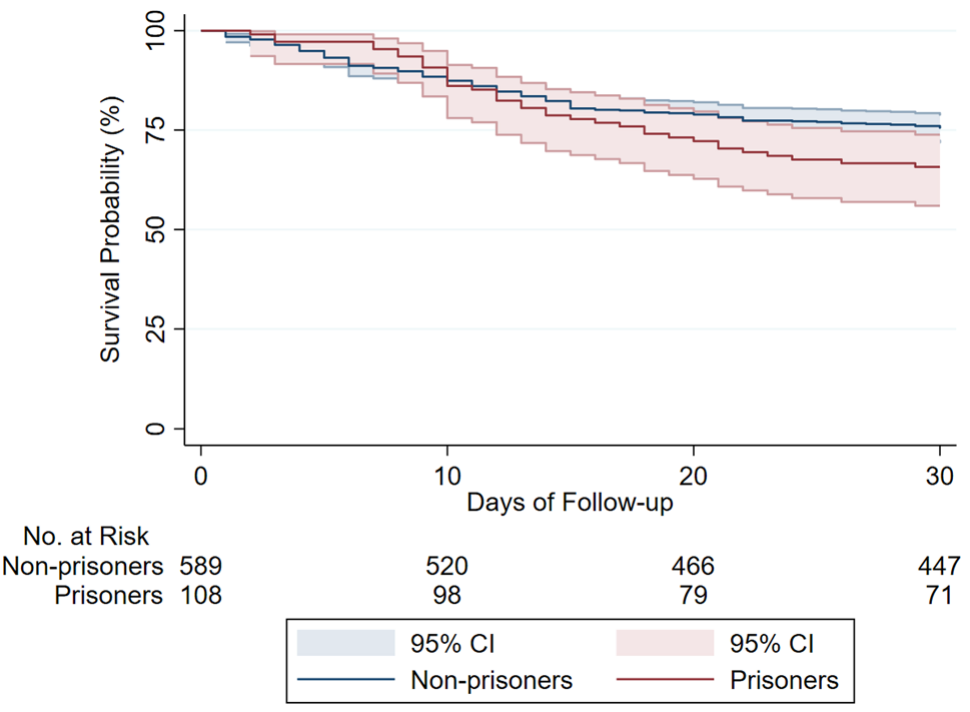
Survival curve for hospitalized COVID-19 positive patients, by imprisonment status. Survival curve shows all-cause mortality for prisoners and non-prisoners (n = 697) hospitalized for COVID-19. Graph is based on a Cox proportional hazards model. Sample size provided reflects 30-day follow-up data availability; data for 30-day mortality was missing for 9 patients from the non-prisoner group. Shaded area represent pointwise 95% confidence interval. Log-Rank test for the survival curve had *p* value of 0.06.

**Figure 2:**
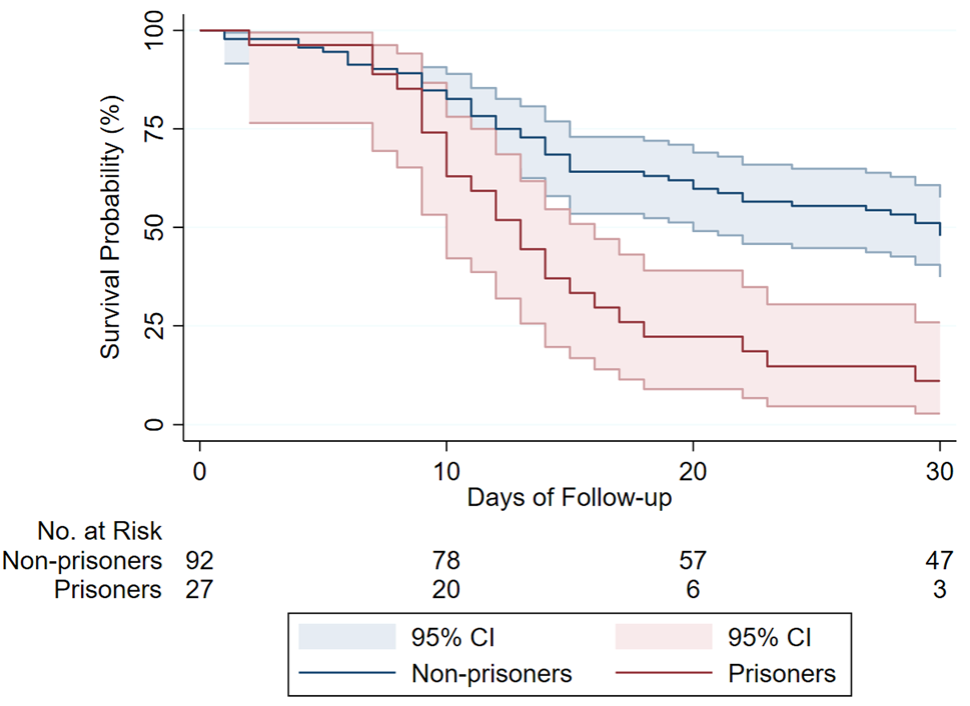
Survival curve for intubated COVID-19 patients, by imprisonment status. Survival curve shows all-cause mortality for prisoners and non-prisoners requiring intubation (n = 119). Graph is based on a Cox proportional hazards model. Data on intubation status was complete for all study participants. Data for 30-day mortality was complete for all intubated patients. Shaded area represent pointwise 95% confidence interval. Log-Rank test for the survival curve had *p* value of < 0.001.

In-hospital all-cause mortality was higher for prisoners compared to non-prisoners (29.6% vs. 20.1%) with an adjusted odds ratio of 2.32 (95% CI, 1.33 to 4.05; *p* = 0.003), Table 3. Mean time from admission to in-hospital death was 12.9 ± 6.6 days for prisoners and 10.4 ± 7.7 days for non-prisoners. Death within 30-day follow-up occurred in 34.3% of prisoners and 24.6% of non-prisoners (Fig. 1). In the adjusted time-to-event analyses (Table 3), prisoners had twice the hazard of 30-day mortality (hazard ratio 2.00; 95% CI, 1.33 to 3.00; *p* = 0.001). Mean time from admission to death was 13.7 ± 6.6 days for prisoners and 11.2 ± 7.5 for non-prisoners. Among patients requiring intubation, 30-day mortality was 59.7% with a striking differential in mortality based on imprisonment status. The 30-day mortality among intubated prisoners was 89% compared to 51% for non-prisoners (Fig. 2), with an age and gender-adjusted hazard ratio of 2.70 (95% CI, 1.54 – 4.68; *P* < 0.001). Variables associated with higher in-hospital and 30-day mortality (Table S4 in Supplement 1) were increasing age, male sex, Caucasian race, and increasing CCI. Imprisonment status was independently associated with increased risk of in-hospital death.

**Table 3:** Clinical outcomes (30-day mortality, in-hospital mortality, and intubation) for hospitalized COVID-19 positive prisoners compared to non-prisoners in the crude analysis (model 1) and multivariable analyses (models 2 and 3).

30-day mortality^a^	HR (95% CI)	*p* value
Model 1	1.41 (0.98–2.03)	0.06
Model 2	1.96 (1.32–2.93)	0.001
Model 3	2.00 (1.33–3.00)	0.001

In-hospital mortality^a^	OR (95% CI)	*p* value
Model 1	1.68 (1.06–2.65)	0.03
Model 2	2.38 (1.37–4.12)	0.002
Model 3	2.32 (1.33–4.05)	0.003

Intubation^a^	OR (95% CI)	*p* value
Model 1	1.86 (1.14–3.03)	0.01
Model 2	1.64 (0.96–2.80)	0.07
Model 3	1.66 (0.96–2.87)	0.07

^a^Model 1: Unadjusted-imprisonment status only model. Model 2: Adjusted for age and sex. Model 3: Adjusted for age, sex, race, Charlson Comorbidity Index (CCI), and obesity.

### Secondary outcomes

A larger proportion of prisoners required admission to the ICU (26.9% vs. 18.7%) and more patients in the prisoners group required pressure support with vasopressors to non-prisoners (24.1% vs. 9.9%). Intubation and mechanical ventilation during hospital course was required in 25.0% of prisoners and 15.2% of non-prisoners (Table 2). In the multivariable logistic regression models, prisoners had higher odds of intubation compared to non-prisoners (odds ratio 1.66; 95% CI, 0.96 to 2.86; *P* = 0.07), Tables 3 and S3 (supplement 1). The median length of hospital stay was 8 (IQR: 5–12) days. For non-prisoners discharged alive, 75.8% were discharged to home and 20.0% to other facilities. For prisoners discharged alive, clinically stable prisoners were discharged to quarantine units within the prison facility (31.6%), while those requiring additional medical care (i.e., high oxygen requirement) were discharged to a prisoner-designated hospital (68.4%), operated by Michigan Department of Correction (Table 2).

## Discussion

Despite more than 300,000 reported cases in the U.S in 2020, no reports to-date have investigated clinical outcomes and mortality trends among prisoners hospitalized with COVID-19. In this study, we report our experience on the comparative outcomes of 108 prisoners and 698 non-prisoners hospitalized with COVID-19. Prisoner status was associated with approximately twice the risk of death compared to non-prisoners. Prisoners in our cohort presented from four local prisoner facilities located in Jackson and Coldwater City in Michigan. In these facilities, positivity for SARS-CoV-2 was reported to range between 38% (708 of 1,872 at one facility) and 55% (793 of 1,430 at another facility)^7^. However, precise estimation for hospitalization rates among COVID-19 positive prisoners was not feasible.

It is well-established that COVID-19 disease passes through three different phases: early infection phase, pulmonary phase, and hyperinflammation phase^10^. The third phase, which is characterized by severe inflammatory response (i.e., cytokine storm), appears approximately 10–12 days after the onset of illness with profound inflammatory profile and worsening hypoxemia^10^. In our cohort, prisoners were more likely to present with features consistent with this hyperinflammatory phase as evidenced by signs of Systemic Inflammatory Response Syndrome (SIRS), markedly elevated inflammatory markers, and severe hypoxemia on presentation. These advanced features argue that the reported time from onset of symptoms to admission for prisoners (median of 6 days) was likely mis-reported in our cohort. Further, the worse clinical outcomes among prisoners are possibly a direct reflection of the late presentation to the hospital from prison facility – whether due to delayed reporting of symptoms by prisoners themselves or higher threshold of referral from prisoner to the hospital than that from the community.

The Michigan Department of Correction have adopted a pre-hospital scoring system to determine referral decisions^11^. The scoring system is based on age, vital signs, oxygen requirements, and mental status (Table S6 in Supplement 1); only prisoners meeting the criteria of the scoring system were considered for transportation to the hospital. On the other hand, the need for supplemental oxygen was the primary deciding factor in admitting COVID-19 patients presenting from the community. Once hospitalized, both prisoners and non-prisoners were managed with similar treatment protocols, irrespective of their imprisonment status. Nonetheless, the pre-hospitalization treatment protocols adopted by the prison facilities remain unknown to us.

A higher percentage of prisoners than non-prisoners required admission to the ICU, pressure support with vasopressors, and intubation. For both in-hospital and 30-day mortality, prisoners had approximately 10% excess mortality rate than non-prisoners. Prisoners who required intubation had strikingly dismal outcomes with 30-day mortality approaching 89%. The discrepancy in mortality rates observed in the study population is multifactorial; while some are attributed to the demographic characteristics and underlying comorbidity burden, others are inherently unique to the imprisonment status. Incarcerated individuals bear a disproportionally higher burden of infectious diseases. Reports have estimated that 25% of all HIV/AIDS cases and 35% of all hepatitis C virus cases in the U.S are among incarcerated individuals^12^. Further, sepsis mortality rates were shown to be four times higher among prisoners to that for non-prisoners (42.5% vs. 15.3%)^13^.

In our cohort, prisoners were younger in age, predominantly males, and had higher prevalence of COPD, diabetes mellitus, underlying malignancies, and a higher CCI score. COPD was overrepresented among patients included in this cohort (14.6%) as compared to a reported prevalence of 2 – 6% in large cohorts from New York, Louisiana, and China^14–16^. Moreover, a disproportionally larger percentage of prisoners had underlying COPD compared to non-prisoners (37.5 vs. 10.5%) – reflecting the higher prevalence of smoking in this group (80.1% were current or former smokers). Data on the association between COPD and disease severity in COVID-19 patients remains scarce. A meta-analysis of limited sample size suggests that COPD patients are at a substantially greater risk of more severe disease and mortality^17^. Nonetheless, the discrepancy in 30-day mortality in our cohort remained remarkable when comparing prisoner to non-prisoner patients without an underlying COPD (29.4% and 23.2%, respectively). Of interest, patients in our cohort have higher prevalence of obesity (defined as BMI ≥ 30 kg/m^2^) in both prisoner and non-prisoner groups (42% vs. 46%). In meta-analysis studies, obesity was strongly associated with poor outcomes in hospitalized COVID-19 patients, including an increased risk for severe disease course, ICU admission, mechanical ventilation, and death. Such high prevalence of obesity adds to the comorbid conditions contributing to the relative high mortality rate in the cohort (30-day all-cause mortality of 26%)^18, 19^.

Studies have determined age to be the most significant risk factor for severe COVID-19 infection^20^. In our cohort, prisoners were proportionally younger than non-prisoners, but interestingly they had worse clinical outcomes overall. Further, the overwhelming majority of prisoners in our cohorts were males (98.2%). Sex-disaggregated epidemiologic data across 38 countries have shown a significantly higher case-fatality rates for COVID-19 among males^21^. Females have stronger innate, humoral, and cellular immunity resulting in sex-specific outcomes from viral infections^22, 23^. In animal models of mice infected with SARS-CoV-1, higher susceptibility to SARS-CoV-1 infection and greater infiltration of neutrophils and macrophages into the lungs were observed in male mice^24^. Further, ovariectomized and estrogen receptor antagonist-treated female mice had increased mortality from SARS-CoV-1 infection^24^. These findings of sex-based differences in immune response and susceptibility can partially explain the excess mortality from COVID-19 among prisoners.

The study has several limitations. First, the observational study design makes selection bias inevitable, especially considering the potential discrepancy in hospital referral and admission threshold between the two study groups. However, our findings remained largely consistent after controlling for potential confounders related to demographics, comorbidities, clinical signs, and markers of disease severity. Second, the study population was limited to one healthcare system in Michigan admitting patients from certain prisons and communities, and therefore external generalizability requires validation. Finally, presented findings were based on data extracted from the electronic medical records of the health system, and hence, is subjected to the accuracy of documentation by the healthcare team, but the outcomes were adjudicated and all patients discharged alive were contacted directly or through their healthcare office.

In conclusion, our study provides insight into the disparity in clinical outcomes among prisoners hospitalized with COVID-19. With COVID-19 becoming one of the leading causes of death in the U.S., findings on the association between imprisonment and COVID-19 mortality are alarming. Responding to the outbreaks in incarceration facilities is uniquely challenging and failure to mount an adequate response to ameliorate COVID-19 outbreaks in U.S prions has the potential to compromise the well-being of prisoners, correctional workforce, and people living in the communities in which prisons are located. Since physical distancing, the most effective mitigative measure, is constantly violated by prisons overcrowding, major shift in policy towards enforcing ‘mass testing and segregation’ and prisons depopulation (i.e., drastically reduce the population of prisoners) might be warranted to abort further imminent outbreaks^1^. Nonetheless, maintaining the rights of prisoners for timely, adequate, and equitable healthcare services is indispensable to close the gap in COVID-19 clinical outcomes with the general population.

## Supplementary Information

Supplementary Information
