# Supplementary material for: Characteristics and comparative clinical outcomes of prisoner versus non-prisoner populations hospitalized with COVID-19
Source: Sci Rep. 2021 Mar 22;11:6488. doi: 10.1038/s41598-021-85916-w (PMC7985211; doi:10.1038/s41598-021-85916-w)
Supplement: Supplementary file 1 — Supplementary Information [file 41598_2021_85916_MOESM1_ESM.pdf]

APPENDIX

Table of Contents

Table S1: Vital Signs and Laboratory Findings in Patients Hospitalized with COVID-19 (Prisoners vs. Non-Prisoners).

Table S2: Complications during hospital course for hospitalized COVID-19 patients.

Table S3: Logistic regression models and odds ratios for intubation.

Table S4: Logistic regression models and odds ratios for in-patient mortality.

Table S5: Cox regression models and hazard ratios for 30-day mortality.

Table S6: MDOC COVID-19 Acuity Scoring system to determine eligibility for transfer from prisoner facility to the hospital.

Characteristics and Comparative Clinical Outcomes of Prisoner Versus Non-Prisoner Populations Hospitalized with COVID-19

Ahmed M. Altibi,[1,2] MD; Bhargava Pallavi,[3] MD; Hassan Liaqat,[1] MD; Alexander A. Slota,[1] MD; Radhika Sheth,[1] MD; Lama Al Jebbawi,[1] MD; Matthew E. George,[4] MD; Allison LeDuc,[1] PA-C; Enas Abdallah,[1] MD; Luke R. Russell,[1] DO; Saniya Jain,[5] MS; Nariné Shirvanian,[1] DO; Ahmad Masri,[6] MD, MS; Vivek Kak,[1†] MD

Affiliations:

1 Henry Ford Allegiance Hospital, Henry Ford Health System, Jackson, MI, USA.

2 Harvard T.H. Chan School of Public Health, Harvard University, Boston, MA, USA.

3 Department of Internal Medicine, Division of Infectious Diseases, Henry Ford Hospital, Detroit and West Bloomfield, MI, USA.

4 Division of Hospital Medicine, Henry Ford West Bloomfield Hospital, West Bloomfield, MI, USA.

5 Michigan State University College of Osteopathic Medicine, Lansing, MI, USA.

6 Knight Cardiovascular Institute, Oregon Health & Science University, Portland, OR, USA.

† **Corresponding Author**

Vivek Kak, M.D., FACP

Department of Internal Medicine

Henry Ford Allegiance Hospital

Henry Ford Health System

205 N East Ave, Jackson, MI 49201 USA

Tel: +1-517-927-3248

Email: Vkak1@hfhs.org

Table S1: Vital Signs and Laboratory Findings in Patients Hospitalized with COVID-19 (Prisoners vs. Non-Prisoners).

	Prisoners (N= 108)	Non-prisoners (N= 598)	*p Value*
**Vital Signs Upon Admission**			
**Respiratory rate >24**	48 (44.4%)	167 (27.9%)	**<0.001**
**Temperature ≥ 38.0 Celsius**	29 (26.9%)	89 (14.9%)	**0.002**
**Oxygen saturation <94%**	52 (48.2%)	223 (37.3%)	**0.03**
**Systolic blood pressure <90 mmHg**	1 (0.93%)	27 (4.7%)	0.08
**Mean arterial pressure (mmHg)** [b]	89.3 ± 16.7	90.1 ± 15.1	0.64
**Laboratory Findings Upon Admission**			
**White blood cell count** [a b]	7.2 (5.4 – 9.2)	6.5 (4.9 – 9.2)	0.27
**Lymphocyte count <1,000/ul**	61 (56.5%)	337/594 (56.7%)	0.96
**Platelet count <150,000/ul**	17 (15.7%)	148/594 (24.9%)	**0.04**
**AST >40 U/liter**[c]	62/106 (58.5%)	271/572 (47.4%)	**0.04**
**ALT >40 U/liter**[c]	30/107 (28.0%)	153/572 (26.8%)	0.78
**Creatinine (mg/dl)**[a]<br>**Creatinine >1.5 mg/dl**	1.6 ± 1.9<br>26 (24.1%)	1.8 ± 6.4<br>160/592 (27.0%)	0.57
**Lactate dehydrogenase** [a b] **(U/L)**	412 (322-547)	288 (218-383)	**<0.001**
**HS-Troponin I >18 ng/L** [c]	38 (35.2%)	212/576 (36.8%)	0.75
**Procalcitonin (ng/ml)**<br><0.25<br>0.25 – 0.5<br>>0.5	 61/106 (57.6%)<br>24/106 (22.6%)<br>21/106 (19.8%)	 361/532 (67.9%)<br>75/532 (14.1%)<br>96/532 (18.0%)	0.06
**C-Reactive Protein (mg/dl)**<br>< 5<br>5 – 10<br>>10	 26/107 (24.3%)<br>27/107 (25.2%)<br>54/107 (50.5%)	 155/559 (27.7%)<br>171/559 (30.6%)<br>233/559 (41.7%)	0.57
**Ferritin (ng/ml)**<br><300<br>300 – 2,000<br>≥ 2,000	 15/107 (14.0%)<br>70/107 (65.4%)<br>22/107 (20.6%)	 181/568 (31.9%)<br>346/568 (60.9%)<br>41/568 (7.2%)	**<0.001**
**D-dimer (ug/ml)**<br>< 0.25<br>0.25 – 0.5<br>> 0.5	 13/105 (12.4%)<br>30/105 (28.6%)<br>62/105 (59.0%)	 58/554 (10.5%)<br>154/554 (27.8%)<br>342/554 (61.7%)	0.63
**Laboratory Findings During Hospital Course**			
**Lymphocyte nadir <1,000/ul**	94 (87.0%)	492/594 (82.8%)	0.28
**Platelet nadir <150,000/ul**	33 (30.6%)	215/594 (36.2%)	0.26
**ALT Peak >40 U/liter**	68/107 (63.6%)	291/571 (51.0%)	**0.02**
**AST Peak >40 U/liter**	74/106 (69.8%)	361/572 (63.1%)	0.19
**HS-Troponin I Peak >18 ng/L**	48 (44.4%)	262/576 (45.5%)	0.84
**Procalcitonin Peak (ng/ml)**<br><0.25	 53/106 (50%)	 340/532 (63.9%)	**0.02**

0.25 – 0.5<br>>0.5	23/106 (21.7%)<br>30/106 (28.3%)	71/532 (13.4%)<br>121/532 (22.7%)	
**C-Reactive Protein Peak (mg/dl)**<br>< 5<br>5 – 10<br>>10	16/106 (15.1%)<br>28/106 (26.4%)<br>62/106 (58.5%)	111/559 (19.9%)<br>128/559 (22.9%)<br>320/559 (57.3%)	0.45
**Ferritin Peak (ng/ml)**<br><300 ng/ml<br>300 – 2,000 ng/ml<br>≥ 2,000 ng/ml	8/107 (7.5%)<br>72/107 (67.3%)<br>27/107 (25.2%)	127/568 (22.4%)<br>369/568 (64.9%)<br>72/568 (12.7%)	**<0.001**
**D-dimer Peak (ug/ml)**<br>< 0.25<br>0.25 – 0.5<br>> 0.5	8/103 (7.8%)<br>24/103 (23.3%)<br>71/103 (68.9%)	32/554 (5.8%)<br>97/554 (17.5%)<br>425/554 (76.7%)	0.53

[a] Data for white blood cell (WBC) count, creatinine, and lactate dehydrogenase (LDH) upon admission were missing for 4, 6 and 35 patients in the non-prisoner group, respectively.

[b] Plus–minus values indicate means ± standard deviation (SD). Values for WBC count and LDH are presented as median (interquartile range).

[c] AST indicates aspartate aminotransferase; ALT indicates alanine transaminase; HS-troponin indicates high-sensitivity troponin.

Table S2: Complications during hospital course for hospitalized COVID-19 patients (n= 706).

	Prisoners (N= 108)	Non-prisoners (N= 598)
**ARDS‡**	40 (37.0%)	176 (29.4%)
**Pulmonary embolism†**	7 (6.5%)	32 (5.4%)
**Deep venous thrombosis**	7 (6.5%)	0 (0.0%)
**Acute kidney injury‡**	49 (45.4%)	247 (41.3%)
**Rhabdomyolysis**	29 (26.9%)	57 (9.5%)
**Ischemic Stroke**	3 (2.8%)	6 (1.0%)
**Myocardial infarction**	2 (1.9%)	4 (0.7%)
**Acute encephalopathy**	4 (3.7%)	29 (4.9%)
**Septic shock**	17 (15.7%)	37 (6.2%)
**Bacteremia¶**	2/30 (6.7%)	22/320 (6.9%)
**Bacterial Pneumonia‖**	8/21 (38.1%)	25/88 (28.4%)
**Influenza A/B**	0/3 (0.0%)	3/151 (2.0%)

† Only pulmonary embolism cases confirmed with imaging modalities (e.g., computed tomography scan) were counted. Suspected pulmonary embolism cases, treated empirically, without a confirmatory diagnosis were not included.

‡ Acute respiratory distress syndrome (ARDS) was defined in accordance with the Berlin Criteria for ARDS. Acute kidney injury was defined in accordance to KIDGO criteria for acute kidney injury.

¶ Blood cultures were obtained in 350 of 706 included patients. Isolated organisms from blood cultures were: staphylococcus aureus (n=7), staphylococcus epidermis (n=5), Corynebacterium (n=5), enterococcus fecalis (n=3), streptococcal species (n=2), and others (n=3). Invasive candidemia was diagnosed in one patient from the non-prisoner group.

‖ Superimposed bacterial pneumonia is confirmed with sputum or tracheal aspirate cultures, which were obtained in a total of 109 patients (21 prisoners and 88 non-prisoners). Bacterial pathogens isolated from sputum/tracheal aspirate cultures were: staphylococcus aureus (n= 16), pseudomonas aeruginosa (n= 12), Escherichia coli (n= 3), klebsiella pneumonia (n= 3), streptococcus pneumonia (n=1) and others (n=9). In addition, candidal species grew in obtained cultures in 6 patients.

Table S3: Logistic regression models and odds ratios for intubation in hospitalized COVID-19 patients (n= 706).

	Model 1	Model 2	Model 3
**Prisoners vs. non-prisoners**	1.86 (1.14-3.03), p= 0.010	1.64 (0.96-2.80), p= 0.070	1.66 (0.96-2.87), p= 0.070
**Age, in 1 year-units**		1.01 (0.996-1.02), p= 0.200	1.01 (0.997-1.03), p= 0.120
**Sex: male vs. female**		1.50 (0.96-2.35), p= 0.080	1.58 (1.00-2.49), p= 0.050
**Race: Caucasian vs. others**			1.01 (0.66-1.53), p= 0.980
**CCI Score**			1.12 (1.01-1.25), p= 0.040
**Obesity: yes or no**			2.05 (1.33-3.16), p= 0.001

***** Results of the logistic regression models are reported as odds ratios (95% Confidence Interval), p-value. Model 1 is **u**nadjusted imprisonment status-only model; Model 2 is adjusted for age and sex; Model 3 is adjusted for age, sex, race, Charlson Comorbidity Index (CCI) score, and obesity (BMI ≥30 kg/m^2). Multiple imputation was used to estimate missing values.

Table S4: Logistic regression models and odds ratios for in-hospital mortality in hospitalized COVID-19 patients (n= 706).

	Model 1	Model 2	Model 3
**Prisoners vs. non-prisoners**	1.68 (1.06-2.65), p= 0.030	2.38 (1.37-4.12), p= 0.002	2.32 (1.33-4.05), p= 0.003
**Age, in 1 year-units**		1.07 (1.06-1.09), p <0.001	1.07 (1.05-1.09), p <0.001
**Sex: male vs. female**		1.94 (1.25-3.0), p= 0.003	1.89 (1.21-2.95), p= 0.005
**Race: Caucasian vs. others**			1.47 (0.97-2.24), p= 0.070
**CCI Score**			1.16 (1.04-1.29), p= 0.006
**Obesity: yes or no**			1.18 (0.76-1.82), p= 0.470

***** Results of the logistic regression models are reported as odds ratios (95% Confidence Interval), p-value. Model 1 is **u**nadjusted imprisonment status-only model; Model 2 is adjusted for age and sex; Model 3 is adjusted for age, sex, race, Charlson Comorbidity Index (CCI) score, and obesity (BMI ≥30 kg/m^2). Multiple imputation was used to estimate missing values.

Table S5: Cox regression models and hazard ratios for 30-day mortality in hospitalized COVID-19 patients (n= 697).

	Model 1	Model 2	Model 3
**Prisoners vs. non-prisoners**	1.41 (0.98-2.03), p= 0.060	1.96 (1.32-2.93), p= 0.001	2.00 (1.33-3.00), p= 0.001
**Age, in 1 year-units**		1.08 (1.06-1.09), p <0.001	1.07 (1.06-1.09), p <0.001
**Sex: male vs. female**		1.75 (1.25-2.44), p= 0.001	1.64 (1.17-2.30), p= 0.004
**Race: Caucasian vs. others**			1.43 (1.04-1.96), p= 0.030
**CCI Score**			1.15 (1.07-1.23), p <0.001
**Obesity: yes or no**			1.03 (0.74-1.45), p= 0.850

***** Results of the Cox regression models are reported as hazard ratios (95% Confidence Interval), p-value. Model 1 is **u**nadjusted imprisonment status-only model; Model 2 is adjusted for age and sex; Model 3 is adjusted for age, sex, race, Charlson Comorbidity Index (CCI) score, and obesity (BMI ≥30 kg/m^2). Multiple imputation was used to estimate missing values.

Table S6: MDOC COVID-19 Acuity Score. Acuity pre-hospital scoring system adopted by Michigan Department of Correction (MDOC), to determine eligibility for transfer of COVID-19 prisoners from the prisoner facility to the hospital.

COVID-19 PATIENT ASSESSMENT								
**Parameters**	**3***	**2**	**1**	**0**	**1**	**2**	**3***	**POINTS**
**Age**				<65			≥65	
**Respiratory Rate**	≤8		9-11	12-20		21-24	≥25	
**Heart Rate**	≤40		41-50	51-90	91-110	111-130	>131	
**Temperature (Oral-F)**	≤35.0		35.1-36.0	36.1-38.0	38.1-39.0	≥39.1		
**Systolic Blood Pressure (optional if 0 points on other data)**	≤90	91-100		101-159		160-179	≥180	
**Oxygen Saturation – Room Air (after >1-minute walking)**	<92*	92-93	94-95	96+				
**Pre-COVID-19 Chronic Supplemental Oxygen**		Yes		No				
**Mental Status**				Normal/Alert			Altered	
**TOTAL POINT SCORE**								

¶ Full score is obtained at least once daily for score 0, and twice daily for score 1-4. Provider was contacted immediately for consideration of transfer to the hospital, if any of the following: oxygen saturation on room air (after >1-minute walking/physical activity) <92%, oxygen requirement is now >2 liter/minute above pre Covid-19 baseline, or patient has three (3) or more points on the scoring system at any measurement (unless the three points are based on age alone).

ǁ Transfer from the prisoner facility to the hospital does not necessarily translate into an admission to the hospital but indicates the need for further evaluation by the emergency department at the hospital to further assess eligibility for admission.
